# A critical systematic review of the Neurotracker perceptual-cognitive training tool

**DOI:** 10.3758/s13423-021-01892-2

**Published:** 2021-04-05

**Authors:** Christian Vater, Rob Gray, Alex O. Holcombe

**Affiliations:** 1 Institute of Sport Science, University of Bern, Bremgartenstrasse 145, CH-3012 Bern, Switzerland; 2 The Polytechnic School, Arizona State University, Mesa, AZ USA; 3 School of Psychology, University of Sydney, Sydney, NSW Australia

**Keywords:** Sport, Transfer, Vision, Attention, Intervention

## Abstract

In this systematic review, we evaluate the scientific evidence behind “Neurotracker,” one of the most popular perceptual-cognitive training tools in sports. The tool, which is also used in rehabilitation and aging research to examine cognitive abilities, uses a 3D multiple object-tracking (MOT) task. In this review, we examine Neurotracker from both a sport science and a basic science perspective. We first summarize the sport science debate regarding the value of general cognitive skill training, based on tools such as Neurotracker, versus sport-specific skill training. We then consider the several hundred MOT publications in cognitive and vision science from the last 30 years that have investigated cognitive functions and object tracking processes. This literature suggests that the abilities underlying object tracking are not those advertised by the Neurotracker manufacturers. With a systematic literature search, we scrutinize the evidence for whether general cognitive skills can be tested and trained with Neurotracker and whether these trained skills transfer to other domains. The literature has major limitations, for example a total absence of preregistered studies, which makes the evidence for improvements for working memory and sustained attention very weak. For other skills as well, the effects are mixed. Only three studies investigated far transfer to ecologically valid tasks, two of which did not find any effect. We provide recommendations for future Neurotracker research to improve the evidence base and for making better use of sport and basic science findings.

**Supplementary Information:**

The online version contains supplementary material available at 10.3758/s13423-021-01892-2.

## Introduction

The primary goal of most types of sports training is to have positive transfer of training to competition. That is, improved performance on game day. Such training includes strength and endurance training, skill training, and perceptual and decision-making training. For the latter, improving perceptual-cognitive skills, (i.e., processing the most important information at the right time to make accurate decisions) likely separates novices from experts (Mann et al., 2007). In this systematic review, we combine research from sports science and basic science to evaluate one of the most popular perceptual-cognitive training tools in sport, the “Neurotracker.”

### Perceptual-cognitive skill training: Specific or general?

Currently, there are two distinct approaches to improve perceptual-cognitive skills. In the first, led mainly by sport scientists, it has been proposed that training should be highly context- and sports-specific. That is, to be effective in improving performance during the actual sport, the training must contain the perceptual information (e.g., spacing between opponents, expansion of a ball) that is present in the actual game (Baker et al., 2003a, 2003b; Broadbent et al., 2015; Williams et al., 2011). This approach dates to Brunswick’s (1956) concept of representative design, which demands representative tasks in perceptual-cognitive skills training that replicate the real world as closely as possible in terms of a few key components (specifically, perception-action coupling, action fidelity, and perceptual information) to improve the transfer of learning (for a discussion, see Broadbent et al., 2015, p. 329).

In the alternative approach, it is proposed that general perceptual and cognitive processes can be trained out of context (e.g., using stimuli and tools from ophthalmology). This type of training is sometimes called brain training, perceptual training, attention training, or mind training (Harris et al., 2018). On occasion, the producers of the associated products have made claims that go beyond that warranted by the evidence. The manufacturers of the Lumosity software, for example, were fined in 2016 for “deceptive advertising” because they suggested that training generic vision and attention skills would help against “memory loss, dementia, and even Alzheimer’s disease” (https://www.ftc.gov/news-events/press-releases/2016/01/lumosity-pay-2-million-settle-ftc-deceptive-advertising-charges, received on 10 August 2020). Recently, Simons et al. (2016) raised serious concerns even about more modest claims for the benefits of brain-training programs after finding low methodological rigor in the studies purporting to show their effectiveness.

A growing body of non-sport-specific vision and attention training techniques are used in the hope that they will improve visual-motor skills in sports (for reviews, see Appelbaum & Erickson, 2018; Hadlow et al., 2018; Harris et al., 2018). The range of training tools spans basic visual abilities such as depth perception and peripheral vision, visual-motor training for eye-hand coordination and other skills, and perceptual-cognitive training for information processing and decision making (for a review, see Appelbaum & Erickson, 2018).

How effective is such general training at improving sport-specific skills? Some recent topical reviews have assessed portions of the existing evidence. Transfer from one task to another has been classified based on amount of difference between tasks (Schmidt et al., 2019) into near transfer (to similar tasks), mid-level transfer (to tasks of a similar cognitive domain), and far transfer (to real-life tasks; Harris et al., 2018; Harris, Wilson, Smith, Meder, & Vine, 2020b). Critically, for sports training, the intention in using a perceptual-cognitive training tool is for it to provide far-transfer effects to improve sport-specific skills on the field. While some are optimistic that such training can transfer to improved performance in competition i.e., “far transfer” (e.g., Wilkins & Appelbaum, 2019), others are skeptical, arguing that such training is only likely to lead to “near transfer” in the form of improvement on the training task itself (Appelbaum & Erickson, 2018; Gray, 2020; Hadlow et al., 2018; Harris et al., 2018; Renshaw et al., 2019).

One of the most popular and well-studied generalized perceptual-cognitive training tools is Neurotracker. Its producers claim that training with it yields benefits that include far transfer. Their home page (https://neurotracker.net/performance/, retrieved 10 May 2020) includes the following phrases that indicate the claims they make for the benefits of Neurotracker training: “focusing on key play opportunities,” “filter out incoming sensory distractions,” “stay sharp under high-pressure demands,” “see more opportunities in any situation,” “interpret body language more effectively,” “perceptively slow down the environment,” “respond more quickly and efficiently,” “improve your response accuracy,” and “avoid overly impulsive actions.” Many of these correspond to far transfer effects, given that the Neurotracker task is restricted to paying attention to moving spheres on a computer display. In this systematic review, we aim to identify what the evidence indicates about the benefits of Neurotracker.

### Neurotracker

Neurotracker is promoted and sold by the Faubert Applied Research Centre with links to the School of Optometry of the University of Montréal, as well as CogniSens Athletics Inc. Professional sports clubs in the NFL, NBA, NHL, and EPL have been reported to use the Neurotracker, as has the U.S. military (https://neurotracker.net/2019/11/27/qa-with-scott-kozak-on-innovations-in-military-training/). Neurotracker is a 3D multiple object-tracking (MOT) task that requires one to fixate on a green dot in the middle of the screen and use peripheral vision to monitor the movements of eight yellow spheres. Each trial consists of four phases, as described by Parsons et al. (2014, p. 4): “During the first phase of each trial, all 8 spheres appear in yellow and are stationary. Next, the 4 target spheres that the trainee must track appear in red for 2 seconds, before switching back to yellow. The spheres begin movement and tracking then occurs over a period of 8 seconds. All 8 spheres move along a linear path through the cube; should any sphere encounter an obstacle it bounces off that obstacle and continues along its new path. At the end of this phase, each sphere is identified with a number and the trainee is asked to verbally state their responses.”

One of the earliest published papers on Neurotracker (Parsons et al., 2014) provides hypotheses regarding potential training and transfer effects, which are frequently cited by Neurotracker proponents. In particular, Parsons et al. (2014, p. 2) claim that the “[ …] cognitive enhancer [i.e., Neurotracker] has four defining characteristics,” although only three are subsequently listed: (1) MOT, (2) large visual field, (3) a binocular 3D display. The Neurotracker, Parsons et al. (2014) state, is based on two principles: “isolation” and “overloading.” Isolation means “that a number of functions solicited for the task should be limited and consistent. A training task should not draw on a random and inconsistent combination of cognitive functions to complete. If isolation does not occur, training effects are reduced... Overloading a function means soliciting it beyond its current ability. To properly train any function, overloading must occur so that adaptation (in the brain: neuroplasticity) can take place.” (Parsons et al., 2014, p. 2). Overloading is achieved by adjusting the speed of every trial to ensure the task is sufficiently difficult.

Parsons et al. (2014) and the Neurotracker website (https://neurotracker.net/benefits/, retrieved 10 May 2020) both state that training with the Neurotracker improves several cognitive functions: attention (sustained, selective, divided, inhibition), short-term memory, working memory and information processing speed (see Table 1). Besides these benefits, Neurotracker is also said to improve “awareness” (e.g., peripheral vision) and decision-making (https://neurotracker.net/performance/, retrieved 10 May 2020). The justification for these claims is not always clear.

**Table 1:** Abilities claimed to be improved by Neurotracker training according to the Neurotracker website

Cognitive function	Definition
Sustained attention	The ability to maintain selective attention over time
Selective attention	The ability to attend to/focus on/cognitively process a given thing
Divided attention	The ability to selectively attend to multiple loci at once (multifocal)
Inhibition	The ability to not attend/focus on/cognitively process a given thing
Short-term memory	The ability to retain information over a short time span (20-30 s)
Working memory	The ability to retain and transform information over a short time span
Processing speed	The time needed to consciously integrate perceptual stimuli

*Note.* Source: https://neurotracker.net/benefits/ (retrieved 10 May 2020). Table adapted from Parsons et al., 2014 who used the definitions from the third edition of the book “Cognitive Neuroscience” by Banich and Compton (2011)

To evaluate the possible benefits of Neurotracker training, this paper will first review work on multiple object tracking generally that has probed its component processes. This first set of work did not investigate the effects of training, but rather used the tools of psychophysics and the experimental study of visual attention to uncover the underlying perceptual, attentional, and cognitive processes involved.

### MOT research and cognitive functions

The first formal study of multiple object tracking was conducted by Pylyshyn and Storm (1988). Participants kept their eyes on a square at the center of the screen (with fixation monitored by an eye-tracker) while attempting to keep track of one to five moving crosses, among a total of ten crosses moving along random paths for 7–15 s. Additionally, they indicated (with a key-press) when any of the target crosses was flashed. If a distractor, i.e., one of the objects that did not need to be tracked, was flashed, the participants were not to respond. Relatively few flash response errors were made (2% for one target, 14% for five targets), showing that participants were able to track up to five out of ten randomly moving objects with high accuracy.

Over the following 30 years, more than 160 peer-reviewed MOT journal articles have been published (Meyerhoff et al., 2017). In their tutorial review, Meyerhoff et al. (2017) explain that it is still unclear whether MOT is a singular process or instead “[…] consists of several subroutines (including attentional selection and working memory processes) that interact with each other based on current task demands" (Meyerhoff et al., 2017, p. 1269). That sentence from Meyerhoff et al. (2017) underscores how much remains unknown about MOT and immediately questions the claim that several abilities are improved by using Neurotracker. However, the basic science of MOT does provide some strong suggestions regarding what processes are involved in Neurotracker task performance.

#### Sustained attention

The Neurotracker website claims that the Neurotracker trains sustained attention, and Parsons et al. (2014, p. 9) specifically argue that 3D-MOT “trains the ability to dynamically shift attention along multiple foci.” However, the authors use this same phrase to define “divided attention” (see “*Divided attention*” section below). In the basic MOT literature, whether or not MOT results in dynamic shifting of attention among the tracked targets has been an active debate since the very first MOT publication, which claimed to rule out shifting of attention (Pylyshyn & Storm, 1988). Some later researchers argued that each object receives its own “spotlight” that works in parallel (sometimes termed multifocal attention; Cavanagh & Alvarez, 2005). More recent evidence, however, seems to indicate that a serial, potentially oscillatory, process imposes the limit on number of targets that can be tracked and maximum tracking speed (Holcombe & Chen, 2013), consistent with recent hybrid models of MOT that involve both parallel and serial processing (Li et al., 2019; Lovett et al., 2019). If these latter theories are correct, then the Neurotracker task should indeed involve the dynamic shift of attention, as claimed, although whether training improves this ability is a separate question.

Accurate performance in the Neurotracker task appears to be limited primarily by how many targets a person can track, and at what speed. MOT research has shown that these two factors, number of targets and maximum speed at which they can be tracked, directly trade off – a person can track a large number of objects moving at a slow speed but only a few objects moving at high speed. If the objects move quickly enough, a person can only track one (Alvarez & Franconeri, 2007; Holcombe & Chen, 2012, 2013). This points to an attentional resource that can be divided among moving objects, and the more attention is divided, the slower a target can be tracked.

A component of attention that limits both number of targets and maximum target speed is, quite surprisingly, specific to each visual hemifield (everything to the left of the point of gaze is the left hemifield, and everything to the right is the right hemifield). That is, if there are enough moving targets confined to one visual hemifield (say, the left one) at a high enough speed that adding an additional target will substantially degrade performance, that degradation does *not* happen if a target is added to the other hemifield (Alvarez & Cavanagh, 2005; Holcombe & Chen, 2012, 2013). If Neurotracker performance is limited by the hemifield-specific resource, it is less likely that the Neurotracker overloads working memory and short-term memory, as they are *not* hemifield-specific (Alvarez et al., 2012).

Sustained attention is expected to be “overloaded” by having objects move in three (3D) rather than two dimensions (2D), because higher speed thresholds can be achieved in 3D (Faubert & Sidebottom, 2012). This expectation is based on the results reported in an abstract to a vision conference (Tinjust et al., 2010). Other MOT studies, however, show the opposite effect: Tracking objects on different depth planes – as in 3D – has been found to be easier than tracking objects on one depth plane – as in 2D (see Cooke et al., 2017; Dünser & Mancero, 2009; Viswanathan & Mingolla, 2002). Similar to 2D MOT, tracking accuracy is impaired in 3D when object speed is increased or when distances between objects are reduced (Cooke et al., 2017; Ur Rehman et al., 2015). Sustained attention involves parallel and serial tracking processes that are sensitive to the number and speed of objects, the distance between objects as well as their location in the visual 3D environment.

#### Selective attention

In the context of MOT, selective attention is the ability to focus on targets rather than distractors and it is expected that higher object speeds and shorter distances between targets and distractors increase selective attention demands (Parsons et al., 2014, p. 9). At the beginning of an MOT task, such as Neurotracker, featural attention (to red, in the case of Neurotracker) is used to select the target objects. Selective attention must then be sustained on these objects when they become identical to the distractors, and then tracked as they move. For neurotypical individuals the initial selection process does not appear to be demanding (Drew & Vogel, 2008) – the average capacity limit for selection is higher than that of tracking (Alvarez & Franconeri, 2007). When the number of objects to track is low and their speed slow, participants can track objects for at least 10 min with little loss (Wolfe et al., 2007). Thus, while many potential athlete users may imagine that a test of attention tests how *long* they can pay attention, this is not likely to be the reason for differences among people on MOT performance. With featural attention (e.g., attention to color) needed for target acquisition unlikely to be taxed in typical people by Neurotracker, and only needed briefly, featural attention seems unlikely to improve with Neurotracker training.

Selective attention is also affected by short-range perceptual interference among targets and distractors (often called “crowding”) when a target gets too close to a distractor (Holcombe et al., 2014; Vater et al., 2017b). There are large individual differences in crowding that correlate with other visual tasks such as spatial localization (Greenwood et al., 2017) and reading (Pelli & Tillman, 2008). Moreover, training on action video games may reduce crowding and improve reading in developmental dyslexia (Bertoni et al., 2019). This raises the possibility that any benefits from MOT training may be due in part, or even in whole, to a reduction in short-range perceptual interference.

For tasks in which participants do not need to keep their gaze fixed on a single location, overt selective attention in MOT can be examined by using eye-tracking devices. The associated studies have found that MOT task participants look some of the time at individual targets, and some of the time at points near the targets’ centroid (i.e., looking at the center of mass between the targets using peripheral vision), even if nothing is there (Fehd & Seiffert, 2008; Lukavský, 2013; Vater et al., 2016, 2017a). Keeping the gaze near the centroid minimizes the average distance into peripheral vision of the targets, which can greatly improve perception of the targets. The proportion of centroid versus target looking depends on the number of targets (Zelinsky & Neider, 2008) and the distance between objects (Vater et al., 2017b; Zelinsky & Todor, 2010). Gaze direction frequently switches among targets (Elfanagely et al., 2011) and is rarely directed at distractors (Fehd & Seiffert, 2010; Lukavský, 2013; Vater et al., 2016, 2017a). When a particular pattern of object trajectories is shown to participants a second time, the gaze pattern tends to be very similar to the first time (Lukavský, 2013). Requiring that participants move their gaze in a specific way impairs tracking performance (Fehd & Seiffert, 2010). It is possible that substantial improvements in performance as a result of MOT training arise from improvements in how selective attention and the eyes are moved, but this does not appear to have been explored.

#### Divided attention

Divided attention is described as the “ability to dynamically shift attention along multiple loci” (Parsons et al., 2014, p. 9), which is exactly the same phrase that the same authors used to describe sustained attention (see section above on “*Sustained attention*”). Another reason the Parsons et al. (2014) definition is inappropriate is that attention to multiple loci may involve simultaneous allocation to multiple loci rather than dynamic shifting (Awh & Pashler, 2000; Pylyshyn & Storm, 1988). In the study the authors referred to when explaining divided attention (Spelke et al., 1976), a dual-task paradigm – with a reading and writing task – was used. Consequently, one can be sure one is studying divided attention with Neurotracker only when it is combined with a secondary task. Otherwise, divided attention cannot be distinguished from selective attention.

One paper on MOT and secondary tasks found that tracking performance is impaired if the secondary task is conversing on the phone, but not if it is a listening task (Kunar et al., 2008). Tracking performance is also impaired when the secondary task is visual change detection (Vater et al., 2017a). These results suggest that visual secondary tasks might impair tracking performance but some auditory secondary tasks may not.

#### Inhibition

According to Parsons et al. (2014, p. 9), inhibition is a process underlying Neurotracker performance and it is “[…] the ability to not focus on non-pertinent information,” which in MOT requires that one “inhibit focus from distractors.” Evidence that distractor inhibition occurs in MOT was found by Pylyshyn (2006) with a probe detection task. Suppression effects depend on the similarity between targets and distractors in their motion and form (Feria, 2012) and depth (Pylyshyn et al., 2008). Relevant to 3D MOT tasks such as Neurotracker, non-targets on a different depth plane have been found to be filtered out *without* the use of inhibition (Pylyshyn et al., 2008). Nevertheless, distractor locations and changes are often perceived (Alvarez & Oliva, 2008; Vater, 2019) and distractor displacements impair tracking performance (Meyerhoff et al., 2015). All of these results indicate that distractor locations are still encoded.

#### Short-term and working memory

Short-term memory as trained by the Neurotracker according to Parsons et al. (2014, p. 9) is described as “the ability to temporarily retain a limited amount of information in consciousness” and working memory as “the ability to manipulate information stored in a temporary bank to suit the task at hand.” These definitions are consistent with large parts of the memory literature (Cowan, 2017).

Parsons et al. (2014) provided no evidence that the Neurotracker trains short-term memory, and in MOT research more broadly, the nature of the link between MOT and memory (short-term or working) is still debated. Studies have found that a concurrent working memory task impairs MOT performance (Fougnie & Marois, 2006, 2009), suggesting processes in common, but this interference may be restricted to spatial memory (Zhang et al., 2010). Potentially, then, spatial memory may be taxed by Neurotracker training. An individual-difference study also found an association between spatial working memory and MOT performance (Wilmer et al., 2016).

#### Information processing speed

Information processing speed is defined by Parsons et al. (2014, p. 9) first as “The time needed to consciously integrate perceptual stimuli” but later as the speed at which visual stimuli enter “bottom-up” through “sensory organs to primary processing areas and then through higher order processing or ‘association’ areas” (p.9). Aside from Neurotracker papers, we did not find any MOT studies purporting to investigate the role of this construct in MOT performance. Parsons et al. (2014) claim that the target speed thresholds measured by Neurotracker “directly evoke visual information processing speed capacities” (p.9). However, the basis for this claim is obscure. Rather than MOT / Neurotracker performance being limited by the speed at which sensory information reaches a particular area of cortex, it may be wholly constrained by other processes, including crowding or the rate at which attention moves or switches among multiple stimuli (Holcombe et al., 2014; Lovett et al., 2019).

Moreover, while Parsons et al. (2014) define information processing speed as having to do with the speed of sensory processing, the tasks they used to assess information processing speed are characterized in the neuropsychology literature as measuring “the efficiency of cognitive function.” It is assessed using timed tests that typically challenge relatively simple cognitive operations (Sweet, 2011). Specifically, the tasks used by Parsons et al. (2014) were subtests of the Wechsler Adult Intelligence Scale, the Integrated Visual and Auditory Continuous Performance Test, and the Delis-Kaplan Executive Functions System Color-Word Interference Test. Performance on these tests may be constrained more by the effectiveness and error-proneness of more cognitive operations than by faster sensory processing (Sweet, 2011).

#### How MOT performance correlates with performance in other cognitive tasks

Studies of individual differences measure the extent to which those who score highly on one task also score highly on other tasks. The results provide an indication of whether the processes that produce variation in tracking performance also produce variation in other specific tasks. Only one published paper, by Huang et al. (2012), tested a substantial number of individuals both on MOT and multiple other attention-involving tasks. Huang et al. (2012) tested approximately 250 people and found that MOT performance correlated highly with several simple tasks requiring judgments about briefly presented visual stimuli. All the tests other than MOT used static rather than moving objects. Positive correlations of between 0.5 and 0.7 with MOT performance were found for visual search for conjunctions, visual search for spatial configurations, counting the number of items in a brief display, identification of a briefly presented post-masked color, symmetry detection, time to make a response to the color of a stimulus, visual short-term memory, and change detection. Much weaker correlations were found between MOT performance and Raven’s test of intelligence (0.27) and also several tasks thought to test suppression or avoidance of interference, such as the Stroop task (0.20), attentional capture (0.02), and inhibition of return (0.08). The findings indicate that only some attention-related tasks cluster together in the variation among individuals, but the full pattern is far from clear.

#### Summary

As can be seen from this review of related MOT research from the last 30 years, it is still debated how the attentional skills, that are claimed to be improved with Neurotracker training, are involved when tracking multiple objects. Whether these skills can be trained with MOT had not been tested when Parsons et al. (2014) made their claims.

### Aims and focus of this systematic review

Since 2014, a number of studies have investigated possible benefits of Neurotracker training. With this systematic review, we aim to provide those using or considering using the Neurotracker with preliminary answers to two questions:

1. Does Neurotracker test and train the cognitive skills that its makers suggest?
2. Do the skills trained transfer to other domains?

To answer these questions, we first evaluate all scientific references provided by the manufacturer and search for additional peer-reviewed journal articles that were not cited. After the search, we discuss the scientific evidence for near or far transfer effects from Neurotracker training studies in different populations and specifically identify cognitive or motor skills that can (or cannot) be improved. Finally, we make suggestions for future research and for practitioners interested in perceptual-cognitive skill training.

## Methods

Major problems for assessing the strength of evidence for claims in psychology include publication bias, researcher degrees of freedom such as not committing to a particular sample size before beginning running participants, and other questionable research practices such as p-hacking. The phenomenon of publication bias is that researchers only tend to publish a study if it favors their hypothesis. This means that effects on average turn out to be much smaller when unpublished studies are included (Ferguson & Brannick, 2012). We contacted Faubert (a co-author on almost every published Neurotracker study) in June 2020 and January 2021 and asked if he knew of any unpublished studies, but we have not received a response. Therefore, we only include studies listed on the webpage or identified by our literature search.

Our literature search consisted of both a search and analysis of the references provided on the webpage (www.neurotracker.net), and also a systematic literature search where we followed the four steps of the *Preferred Reporting Items for Systematic Reviews and Meta-Analyses* (PRISMA) statement (Moher et al., 2009): identification, screening, eligibility inspection, and inclusion of relevant papers. After the identification stage, the reference lists from both the webpage and the search were combined and checked for duplicates before continuing with the subsequent PRISMA steps of screening, eligibility inspection and inclusion.

### Identification of studies

The Neurotracker webpages link to a document with 35 summaries of studies completed (https://drive.google.com/file/d/11opgnL6lRmnlkW-pNmhqdB_6BZpLp52O/view, retrieved 10 May 2020). On the first pages of the document, each reference is linked to a slide, where the aims, methods and findings of the research are summarized. In some cases, illustrations of the study or results are displayed on the slides too. Two of the 35 research items are linked to the same slide (study #18 and #19 and #27 and #30), so it appears the list actually consists of only 33 research items. A web-link to each research item is included, allowing us to classify items as “peer-reviewed journal articles,” “journal articles without peer-review,” “preprints,” “conference abstracts,” and “other.” An additional Google Scholar search using the title of each item was conducted to check whether the research was published elsewhere. If a research item was presented at a conference or listed as a preprint, but was eventually published in a journal, the reference is here listed as “journal article.” The list of references is likely complete, or nearly complete, as the latest was published on 17 April 2020 (Lysenko-Martin et al., 2020).

**Fig. 1:**
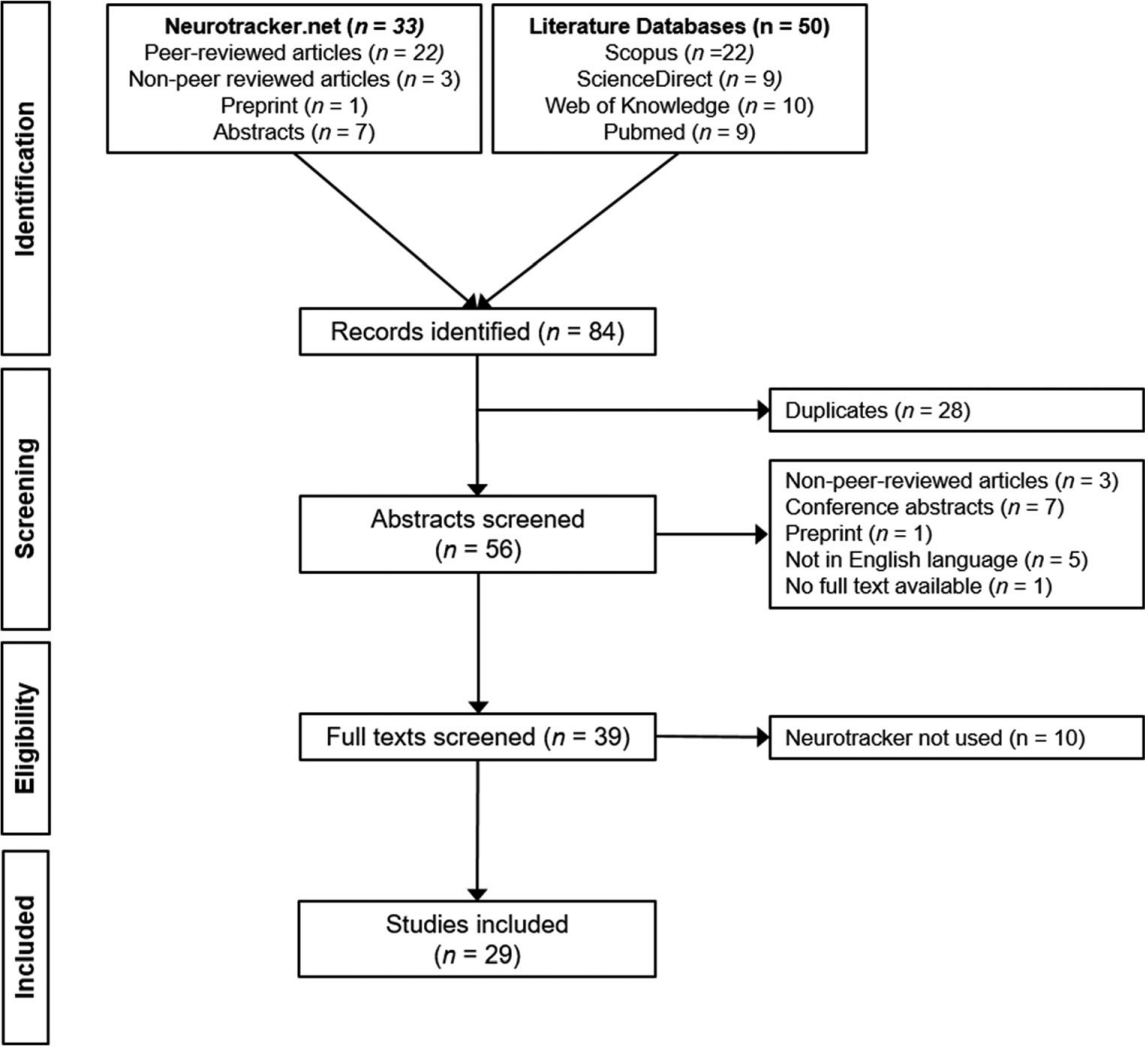
PRISMA scheme for the identification, screening, eligibility, and inclusion stages

In a second identification step, we conducted a systematic literature search in May 2020 using the Scopus, ScienceDirect, Web of Knowledge, and Pubmed databases. The searches were conducted by two raters, the first author and a trained student assistant, working independently. In each database, we searched for the word “Neurotracker” in “all fields” and limited the results to English and to the type “article.” Any conference abstracts, dissertations, book chapters, and reviews were thereby excluded. The results were exported as .ris, .bib or .nbib-files and imported into the citation software ®citavi (2018; version 6). With this procedure, we identified 50 articles (Fig. 1).

### Screening, eligibility, and inclusion

After combining the references of both searches, 84 research items were searched for duplicates, and using the built-in function of citavi, the 28 duplicates were removed. Next, we continued with screening to limit the set to articles published in peer-reviewed journals. We excluded articles that were not published in a peer-reviewed journal (3), as well as conference abstracts (7), preprints (1), articles that were not in English (5), and articles with no available full text (1). After removing these 17 research items, 39 full texts remained. We excluded ten studies that did not use the Neurotracker in their experiments. These papers mainly cited a paper with “Neurotracker” in the title, so that the search criterion was only found in the reference list. In the end, 29 papers could be included in this systematic review.

### Data extraction and analyses

The information extracted from each article followed criteria similar to those of Simons et al. (2016),^1^ adapted for the current review (see Table 2). With this extracted information, we first provide an overview of experimental designs and findings of all the studies.

**Table 2:** Information extracted from each study

Category	Sub-category	Definition or example
Study Information	Source	Reference from homepage or literature search
	Title	Article title
	Authors	List of authors
	Year	Year of publication
	Journal	Journal name
	Peer-reviewed	Yes/no indicating whether the journal includes peer review
	Design	Experimental intervention, correlation assessed, between-group comparison, technical report, theoretical report
Intervention group	N training	Number of participants in the training group
	Participant type	Researched population
	Age group	Mean or range of age of participants
	Intervention interval	Number of training/test sessions (and distribution over time)
	Total training time	Number of minutes (estimated based on number of sessions)
	Training task	Task used in intervention group
Control group	N control	Number of participants in the control group
	Type of control	None, active, passive, placebo
	Matched control	Criteria on which participants were matched between groups
	Participant type	Researched population
	Age group	Mean or range of age of participants
	Control interval	Number of training/test sessions (and distribution over time)
	Total training time	Total minutes (estimated based on number of sessions)
	Training task	Task used in control group
Methods	Nr. Targets	Number of MOT targets
	Nr. Distractors	Number of MOT Distractors
	Outcome task	Task with main dependent variable
	Additional tasks	Other tasks with dependent (or control) variables
Results	Measurement*	Indicates if Neurotracker was used for measurement
	Learning*	Indicates if participants improved Neurotracker performance
	Transfer*	Indicates if Neurotracker training improved other skills
	Near transfer	Indicates improvements in a similar task
	Far transfer	Indicates improvements in a real-world task
Trained skills	Attention*	Improvements in selective attention, divided attention, sustained attention, or short-term memory
	Awareness*	Improvements in perception
	Decision-making*	Improvements in decision-making
	Executive function*	Improvements in inhibition, shifting, or switching
	Working memory*	Improvements in working memory
	Processing speed*	Improvements in processing speed

*Note*. The symbol “*” indicates items that are included in the overview of references on the Neurotracker webpage

In the next step, we evaluate the intervention studies for possible transfer effects. To do this, we check whether the Neurotracker was used for measurement (M), to assess learning effects (i.e., improved Neurotracker performance with practice) (L), and whether a study investigated transfer effects (T) to another task. This trichotomy was used by the manufacturer for their list of references.

Similar to previous reviews (Harris et al., 2018; Simons et al., 2016), we distinguish between near transfer effects (to a similar task) and far transfer effects (to a real-life task). In the summary table (Table 5), we use colors to indicate whether transfer tests yielded positive results (green), positive but with methodological concerns (yellow) or no effect (red). The evaluation of the methods is based on criteria proposed by Simons et al. (2016) and described in more detail in our section “Intervention studies and transfer effects.”

## Review and discussion

On the webpage “Neurotracker.net,” 33 research outputs are provided as scientific references. These references consist of 21 peer-reviewed articles, three articles published in journals with no peer-review, one preprint, seven conference abstracts and two items that could not be found after the additional online search. From the 21 peer-reviewed articles, one was published in Spanish language (Junyent et al., 2015) and was therefore excluded. With our literature search, nine additional articles could be identified that used Neurotracker, but the full text of one study was not available (Varanoske et al., 2020). Thus, our evaluation was based on 29 published studies (see Table 3).

**Table 3:** Included studies with first author, year of publication, study design, any outcomes other than Neurotracker performance, and the main claimed findings

First author	Year	Design	Additional tests	Main claimed finding(s)
Assed et al.	2016	Intervention	Memo Checkup	Neurotracker training led to improvements in episodic and working memory, faster information processing speed, a reduction in complaints, and an improvement of quality of life
Chamoun	2017	Between-group	Motion and orientation discrimination task.	No effect of pharmacological manipulations of cholinergic neurotransmission on Neurotracker performance compared with a placebo. Young adults improved their Neurotracker performance
Chermann et al.,	2018	Within-between-group	SCAT (concussion) and M-BESS (balance)	Athletes have impaired Neurotracker learning rates after injury Performance was correlated with the number of symptoms, SAC- and M-BESS scores 48 hours after injury
Corbin-Berrigan et al.,	2018	Intervention		Individuals with mTBI showed smaller training gains at visit 2 than healthy controls, but the groups did not differ on the remaining visits
Corbin-Berrigan et al.,	2020a	Intervention	Balance and Coordination evaluation; Self-reported fatigue; Self-efficacy on athletic skills and mTBI presentation related to physical activity; computerized cognitive test battery	Clinically recovered mTBI patients improved Neurotracker performance with training but there was no transfer to balance, coordination, self-efficacy, fatigue, or cognitive efficiency
Corbin-Berrigan et al.,	2020b	Intervention	Balance test (BESS); Self-Efficacy, ImPACT, PCSI	Symptomatic children after mTBI can safely perform Neurotracker training. Self-reported fatigue (*p* = .05) and possibly cognitive efficiency (*p* = .08) improved, but there was no change in coordination, balance, self-efficacy or parent-reported quality of life, and no non-Neurotracker comparison group
Fabri	2017	Within-between-group	Postural stability on different surfaces	Older children perform better than younger children in Neurotracker. For both groups, Neurotracker can be combined with a postural stability task without performance impairments
Faubert	2012	Theoretical paper	-	Predicts that Neurotracker training will increase in-field performance in sports, improve collision awareness and that it will be proved useful for concussion assessment
Faubert	2013	Between-group	-	Professional athletes, high-level amateurs, and non-athlete university students significantly differ in Neurotracker learning
Fragala	2014	Intervention (with resistance training)	Visual reaction time (Dynavision D2) and blood parameters (BDNF)	Resistance training might preserve or improve spatial attention and reaction time with aging
Harenberg	2016	Correlation	Laparoscopic surgery task	Neurotracker performance correlates positively with simulated laparoscopic surgery performance
Harris	2020a	Between-group and intervention	MOT, n-back task	Undergraduate students show neither near transfer (2D MOT) nor far transfer (route monitoring task) but did improve working memory performance
Harris	2020b	Intervention	MOT, n-back task, concurrent route recall and auditory monitoring task (real-world military task)	Undergraduate students show Neurotracker learning effects and improvements in a working memory transfer task
Legault	2012	Intervention	Biological motion task	Biological motion perception improved with Neurotracker training at 4-m viewing distance, but not at 16 m
Legault	2013	Between-group and intervention	-	Older adults show slower tracking speeds than younger adults in the four-target condition and younger adults have overall higher speed thresholds
Lysenko-Martin	2020	Correlation	Diagnostic for post-concussion syndrome (PCS); SCAT (concussion)	Neurotracker performance in under 13-year-olds with a concussion history is positively associated with cognition and balance and negatively associated with concussion symptom severity. Males show better Neurotracker performance than females
Mangine	2014	Correlation	Game statistics from season; D2 for visual motor reaction time	NBA point guards and shooting guards possess a faster Neurotracker speed threshold than players from other positions. NBA performance (steals, turnovers, assists) is associated with Neurotracker performance
Michaels	2017	Correlation	Driving task	Neurotracker performance is associated with elevated crash risk and with decreased driving speed, particularly among older adults
Mejane	2019	Within-group comparison	Jumping task (knee rotation)	Neurotracker has no significant effect on knee rotations, either pre- or post-fatigue. A subgroup of 12 athletes showed a significant increase in knee abduction when tested simultaneously with Neurotracker, only in the fatigued condition
Moen	2018	Intervention	Attention network test; Anti-saccade task; Color-shape-task; Letter memory task	Athletes from different sports show Neurotracker learning effects but no transfer effects to executive functions
Musteata	2019	Intervention	Verbal Learning Test (Episodic memory), Digit Span (working memory), D-KEFS Trail Making Test (processing speed, motor speed, cognitive flexibility), D-KEFS Verbal Fluency Test (processing speed, cognitive flexibility), Stroop Test (selective attention, psychomotor speed, cognitive flexibility)	Older adults show Neurotracker learning effects and transfer effects to memory and working memory tasks. Positive transfer was also found for cognitive flexibility and processing speed
Parsons	2016	Intervention	IVA+Plus CPT, WAIS-III subtests: symbol; search, code, block design, number sequence, letter-number sequence and spatial span; d2 attention test; D-KEFS	Neurotracker training can improve attention, visual information processing speed, and working memory, and also leads to changes in resting-state neuroelectric brain function
Plourde	2017	Within-between-group	-	Stereopsis boosts performance on the Neurotracker task in children and adults, but has no impact on older adults’ performances
Romeas	2016	Intervention	Soccer field test	Decision-making accuracy in passing, but not in dribbling and shooting of university-level soccer players is improved with Neurotracker training
Romeas	2019	Intervention	Biological motion perception task	Consolidated Neurotracker training (i.e., training with Neurotracker first and the motor or perceptual task thereafter) leads to better Neurotracker performance than simultaneous Neurotracker training when combined with a motor task but not when combined with a perceptual (biological motion perception) task
Tullo	2018a	Correlation	WASI-II	Neurotracker performance is positively associated with fluid reasoning intelligence
Tullo	2018b	Intervention	CPT-3; WASI-II; FSIQ derived from verbal and non-verbal subtests included in the respective Verbal Comprehension Index (VCI) and Perceptual Reasoning Index (PRI)	Neurotracker training improves CPT-3 performance (rapid response to flashed letters, non-response to ‘X’) in school-age children with neurodevelopmental conditions
Vartanian	2016	Intervention	Shipley-2 working memory span tasks	Members of the Canadian Armed Forces show significant gains in working memory span (verbal, visual, and matrix span) after Neurotracker training

*Note*. Studies are sorted in alphabetical order. Please note that the findings reported here are those claimed by the authors. In some cases, as discussed later, the findings are questioned due to methodological concerns (see Table 5 and the discussion thereafter). Abbreviations: CPT-3 = Conners Continuous Performance Task; D-KEFS = Delis-Kaplan Executive Functions System Color-Word Interference Test; FSIQ = Full Scale Intelligence Quotient; ImPACT = Immediate Post-Concussion Assessment and Cognitive Testing; IVA+Plus CPT = Integrated Visual and Auditory Continuous Performance Test; M-BESS = Modified Balance Error Scoring System; mTBI = mild traumatic brain injury; PCSI = Post-Concussion Symptom Inventory; SCAT (SAC)= Standardized Assessment of Concussion; WAIS = Wechsler Adult Intelligence Scale; WASI-II = Wechsler Abbreviated Scale of Intelligence – Second Edition

Since 2012, articles using Neurotracker have been published in internationally well-known journals such as *Scientific Reports*, *Psychology of Sport and Exercise*, *Intelligence,* and *Frontiers in Psychology*, while others were published in less well-known journals, such as the Brazilian journal “Dementia & Neuropsychologia” and “Ageing Science & Mental Health Studies” on the Research Open platform. The three most cited articles on Google Scholar and Scopus (date of search: 5 May 2020) are:

1. Faubert (2013, Scholar: 162 citations; Scopus: 78 citations): This study compared professional athletes, elite amateurs, and non-athletes on Neurotracker learning rates.
2. Faubert and Sidebottom (2012, Scholar: 145 citations; Scopus: 60 citations): a theoretical article discussing the potential benefits of Neurotracker training.
3. Romeas et al. (2016, Scholar: 130 citations; Scopus: 63 citations): an intervention study that claims that Neurotracker training improves decision-making for passes in soccer.

In terms of the study designs, the included studies consist of 17 intervention studies (i.e. studies that used Neurotracker as a training tool for more than one training session), all of which used the Neurotracker as the intervention, except Fragala et al. (2014) which used a resistance-training intervention and Neurotracker to measure performance in the pre- and posttest; we report the findings of that study only in the Neurotracker non-intervention study section in the supplement section. The remaining sixteen comprise five correlational studies, three within-between-group comparisons, two between-group comparisons, one within-group comparison, one study with a combination of a between-group comparison (Experiment 1) and an intervention (Experiment 2) and one theoretical paper (the Faubert & Sidebottom paper).

In the following, to assess the most important issue of whether there are near or far transfer effects, we will focus on the intervention studies. The other sorts of research designs in this literature typically do not provide good evidence for causal effects of training (Shadish et al., 2002, p. 484) – while quasi-experiments may provide good evidence, this literature contains only simpler observational studies. To provide a complete overview of the literature, however, we also summarize the non-intervention studies in the Supplement section.

### Intervention studies

In this section we will evaluate the scientific quality of the intervention studies. As Simons et al. (2016) argued for studies of “brain training” products, the inclusion of appropriate control groups, ideally “active” controls with similar demand characteristics to the treatment group, are critical to understand the training and transfer effects of an intervention. It may not be possible to always follow best practices in a study because of their applied nature (e.g., in the sports context). Nevertheless, including a control group that did *not* train with Neurotracker and adding a transfer task can be seen as the minimum requirement to garner quality evidence regarding whether Neurotracker training improves another skill. Of the 16 intervention studies, ten studies fulfilled these two criteria while six studies did not. In Table 4, we indicate the extent of transfer effects on attention, awareness, executive function, working memory, processing speed (all near transfer), and decision-making (far transfer), first for the ten studies which fulfilled the two criteria (black font) and thereafter for those that did not (grey font).

**Table 4:**
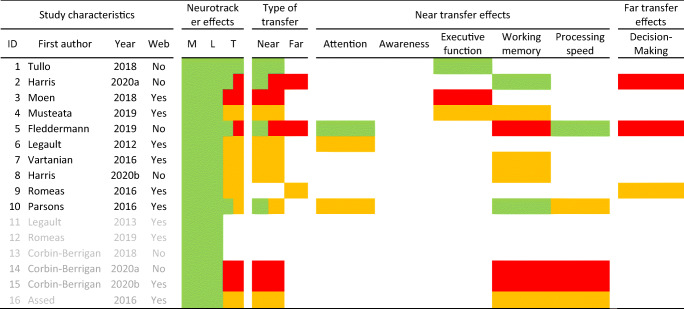
Intervention studies and their characteristics

*Note.* Studies are sorted by the number of participants in descending order (the same study IDs are used Tables 5 and 6). Web – indicates whether the reference is included at Neurotracker.com. Neurotracker effects (M = “Measurement,” L = “Learning,” T = “Transfer”; see Table 2 for definitions), Type of transfer (Near – to a similar task or cognitive domain, Far – to a real-life task) provides a summary of the subsequent columns: trained skills (attention, awareness, decision-making, executive function, working memory, and processing speed). Colors: green = positive effects; yellow = positive effects with methodological concerns; red = no effects; blank = not tested. Studies highlighted with grey text are not counted as intervention studies as they did not include a transfer task (studies 11*–*13) and/or did not include a control group (studies 14*–*16)

In the following, we summarize the results of each of these 16 studies and discuss whether certain methodological concerns apply. Simons et al. (2016, p. 171ff.) criticized studies that have small sample sizes, are not preregistered (with an analysis plan including explanations how variables are coded and analyzed), are suggestive of benefits without mentioning negative consequences, omit adequate controls for placebo effects, have passive or active but unmatched control groups (matching control groups is required to “equate for all aspects of the intervention other than the hypothesized critical ingredient, including expectations to the extent possible”), do not have random assignment to conditions, do not fully report and analyze outcome measures, are not independent from other studies, assess benefits only for the trained task or very similar tasks, rather than assessing transfer, rely on secondary analyses that should be treated as exploratory, or report that interventions work by analyzing only a subgroup of participants.

#### Intervention studies meeting the minimum quality criteria

##### Study 1

In children with neurodevelopmental disorder (e.g., with ASD or ADHD), Neurotracker training was found to improve performance withholding of responding when an ‘X’ was presented but pressing a key when another letter was presented (the CPT-3 Conners Continuous Performance Test) (Tullo, Guy, Faubert, & Bertone, 2018b). 129 participants from elementary and secondary schools were assigned to three groups: a Neurotracker intervention group (Neurotracker), an active control group (computer game) and a passive control group. The training duration was 5 weeks. The groups were matched on age and two WASI (Wechsler Abbreviated Scale of Intelligence) intelligence sub-scales. The Neurotracker group improved significantly more than the control group (which did not improve at all) in Neurotracker and CPT-3 performance indicating a near-transfer to executive functions.

##### Study 2

No transfer effects were found from Neurotracker training to a 2D-MOT task or to a simulated driving task, but improvements were found for a working memory task (Harris et al., 2020b). Eighty-four participants were randomly assigned to a passive control group or one of three Neurotracker training groups (five or three large screen sessions or five small tablet sessions). All groups were tested in near transfer tasks (2D-MOT, working memory) and a far transfer task (a route recall task used in military settings). The results showed significant Neurotracker learning effects in all intervention groups and a marginally significant learning effect in the control group (*p* = .051, *d* = 0.45). For the 2D-MOT task, all groups significantly improved performance from pre- to post-test, with no significant different in amount of improvement. For the working memory task, significant improvements were observed for the full, portable and abbreviated training but not for the control group. There was no time or group effect for the far transfer task. The authors were surprised by not finding a near transfer effect to 2D-MOT and state: “If any transfer effect from Neurotracker training does exist in this case, it is much smaller than the improvement from repeating the MOT test.” (Harris et al., 2020b, p. 5). A limitation was acknowledged by the authors for the working memory measure. As in their previous study, groups were not well balanced at pre-test, so that, for example, the abbreviated three-session group was no better than the control group at the post-test. A further limitation is that the driving task has not previously been validated and may not be a fair test of Neurotracker.

##### Study 3

Moen et al. (2018) found, for elite athletes in several sports, that Neurotracker training did *not* improve executive function. Four executive function tests were used: an attention network test, anti-saccade task, color shape task and letter memory task. Two groups were compared, one with athletes from wrestling, handball, biathlon, and alpine skiing and one with athletes from soccer, paralympic sports, boxing and orienteering. The authors used a cross-over design so that both groups acted as an intervention and as a passive control group once. After each intervention phase, changes in executive functions were examined. The intervention groups showed no differences compared with the control groups in executive functions. In both intervention phases, the intervention groups improved their Neurotracker performance over time. The authors concluded that Neurotracker may not be appropriate to improve specific executive functions. Note, however, that there was a wide range of training sessions in both groups (between nine and 76 training sessions in group 1 and between 14 and 61 sessions in group 2) and a variable number of targets to track (between two and four). Thus, training duration and tracking difficulty were different between groups, which could have affected the average performance in both groups.

##### Study 4

Musteata et al. (2019) found Neurotracker transfer effects in long-delay recognition memory performance, cognitive flexibility and selective attention in older adults. In their study, 25 older adults received 14 Neurotracker training sessions over seven weeks. Participants in the intervention and the passive control group (*n* = 22) underwent some cognitive tests (executive functions and working memory; 18 different variables) before as well as one and five weeks after the intervention. One week after training, the intervention but not the control group showed improvements in working memory (*p* = .01, partial eta squared=.138; California Verbal Learning Test Second Edition) and executive functions (i.e., category switching, *p* = .050, partial eta squared=.083). Improvement in the Stroop inhibition task (*p* = .050, Partial Eta Squared =.082) was reported, but this effect was only found in the “OFF” version of the task (i.e., when the examinees name the color of the ink of a set of number signs). The two claimed “*p* = .050” results seem, however, to reflect erroneous statistical reporting. The *p* value for the OFF Stroop test was reported as .050, the *F*-statistic reported in the associated table, 4.002, seems to instead correspond to *p* = .056. The *F* statistic reported in the text, as opposed to the table, is different (4.065), which corresponds to *p* = .055, assuming that the denominator degrees of freedom given (25) were correct and the numerator is 1 as it is a simple contrast. And for the executive function test reported as *F*(25) = 4.065, *p =* .050, instead *F*(1,25) = 4.065 corresponds to *p* = .055. Additionally, correction for multiple comparisons would have been in order as there were 18 cognitive sub-tests investigated. Five weeks after the intervention, none of these group differences were statistically significant anymore. Instead, other effects (e.g., episodic memory) were observed and it was explained that Neurotracker training could lead to some “delayed effects.” Based on the episodic memory effect 5 weeks after training, the authors state that Neurotracker intervention “[…] may play a significant role in dementia prevention or cognitive decline but further research is needed to ensure reliability and validity” (Musteata et al., 2019, p. 12). A strength of this study is that all cognitive variables were comparable at pre-test for the two groups. At least two of the reported statistics were apparently not reported correctly, however. The study results suggest that, even if there are working memory and executive function effects, they are no longer visible 5 weeks after an intervention. Since it was not controlled what participants did after the intervention, the effects observed 5 weeks post-intervention should be interpreted more cautiously.

##### Study 5

Neurotracker seems to lead to improvements in processing speed and sustained attention in volleyball experts, without significant working memory improvements or far transfer effects (Fleddermann et al., 2019). The intervention group received 8 weeks of Neurotracker training with two sessions per week and was compared to a control group that received only regular volleyball training. Neurotracker performance, memory span, working speed, sustained attention and processing speed were compared between groups. A far-transfer task of physically jumping (block jumps) under single and dual-task conditions was included. The “dual-task high” block jump condition may potentially mirror some of the demands of Neurotracker because the participants had to monitor the movements of a (video-recorded) attacking player with their peripheral vision and perform a maximum block action to the right or left depending on the movement direction of the attacking player. The Neurotracker group improved their Neurotracker performance, in contrast to the control group. The Neurotracker group also showed improvements in sustained attention and processing speed (near transfer). In the far transfer task, response accuracy was over 95% for pre- and posttest in both groups and the main dependent measure, jumping height, showed no differences between groups (Fleddermann et al., 2019, p. 1599). Study strengths are its fairly large sample size of high-level athletes, including a control group, although the intervention group likely had a greater expectation of improvement.

##### Study 6

Results from Legault and Faubert (2012) suggest a transfer effect of Neurotracker training to biological motion perception in older adults. A Neurotracker group trained on Neurotracker in five 30-min sessions while for a control group, the five sessions consisted of training on recognizing the orientation of a simple stimulus at progressively (controlled by a staircase) lower contrast. After the last session, both groups were tested in a task to discriminate the motion direction of a point-light figure, which was masked by noise dots. The dependent variable was the tolerable noise quantity (the more the better). After the training, more noise dots could be tolerated by the Neurotracker group compared with the control group but only for one of the two simulated viewing distances (*p* = 0.04). The control group did not become better in the contrast task over the course of training. Comparing an intervention to an active and passive control group is one of the strengths of the study. The choice of the control task, however, seems not to be a fair comparison to the intervention group, because Neurotracker training improves the tracking of targets amidst distractors, a skill which is also needed in the transfer task, where the noise dots were distractors. Thus, the kind of transfer is difficult to classify here. The contrast task did not involve motion perception or demand sustained attention. Also, the authors did not provide information on performance in the pre-test, so it remains unclear whether the Neurotracker group improved more on the motion task.

##### Study 7

Working memory performance improved in a military population after Neurotracker training (Vartanian et al., 2016). The study assigned members of the Canadian Armed Forces (age 21–50 years) to an intervention group (*n* = 13), active control (*n* = 13), and passive control (*n* = 14) group. The intervention group received 10 min of Neurotracker training ten times over 2 weeks. The active control group was trained in a working memory task: an adaptive dual auditory-visual n-back task. The results indicate that Neurotracker performance improved with training. The Neurotracker training group also improved in word span (*p* = .005, *d* = .96), visual span (*p* = .050, *d* = .60) and matrix span (*p* = .015, *d* = .79) while for the active control group the improvemnets did not reach statistical significance: word span (*p* = .056, *d* = .56), visual span (*p* = .057, *d* = .58) and matrix span (*p* = .180, *d* = .39). The passive control group showed a trend for improvements in visual span (*p* = .198, *d* = .45) and matrix span (*p* = .115, *d* = .49). While similar improvements for visual span were observed in the active control group and Neurotracker group, verbal and visuospatial working memory capacity improved statistically significantly only in the Neurotracker group. To show a training benefit relative to the control group, however, improvements in training group must be compared directly to the improvements in the control group, which was not reported. A direct statistical comparison of the improvements between those two groups is unlikely to have been significant.

##### Study 8

Neurotracker training may improve working memory in university students (Harris, Wilson, Crowe, & Vine, 2020a, b). In Experiment 2 of this study, 36 participants (no specific sports expertise mentioned) were randomly assigned to a Neurotracker intervention or a passive control group. The intervention group received five sessions of Neurotracker training. Both groups were tested in a pre- and post-test in 2D-MOT and a working memory task (n-back task). The Neurotracker group improved their Neurotracker performance more than did the control group. These improvements did not transfer to advantages in the 2D-MOT task, where performance and gaze behavior during the post-test was not different between groups. The Neurotracker group improved their Neurotracker and working memory performance from pre- to post-test. The improved working memory performance was, however, not different to the control group in the post-test. The authors explain in their limitation section that the groups were not equivalent in working memory performance before training and even the control group had a trend for improvement in working memory performance (*p* = 0.08, *d* = .443, *BF*10 = 1.03). The comparison of performance in the 2D-MOT and Neurotracker has to be interpreted with caution, as objects moved on straight paths in the former and random motion paths in the latter. Randomly moving objects are probably more difficult to track. Nevertheless, one might expect that performance might transfer from a more difficult tracking task (Neurotracker) to the likely easier task (2D-MOT).

##### Study 9

Neurotracker training could potentially lead to improved decision-making in passing accuracy in soccer (Romeas et al., 2016). The intervention group (*n* = 7) consisted of university-level soccer players who received ten Neurotracker training sessions (two per week). Data of an active control group (*n* = 7; watched 3D soccer videos in ten sessions) and a passive control group (*n* = 7) were collapsed in the analyses (*n* = 12; two participants were excluded due to injuries). In a field test, all participants from all groups were randomly distributed to teams and played 5 x 5 soccer matches on a 30 m x 40 m interior turf soccer field. Decision making accuracy of passing, dribbling and shooting was analyzed with “standardized coding criteria” by one experienced soccer coach (“objective decision-making assessment”), who was blinded to the experimental protocol. Also, subjective ratings of the players’ decision-making (rated from 0% to 100%) were collected at pre- and post-test (“subjective decision-making assessment”). The coach’s assessment indicated improved decision-making accuracy in passing (+15%), but not for dribbling and shooting, when comparing Neurotracker with the control group. The subjective confidence ratings were higher in Neurotracker compared with the control group. Rating by a single coach, however, has limited validity. Typical sports training studies using such assessments typically have at least two raters and assess inter-rater reliability (see, e.g., Roca et al., 2013). In general, there was no theoretical prediction that decision-making in one or all of the soccer skills would be improved. That there was no effect for dribbling and shooting could simply be explained by the low number of passes and shots that were observed (this point is also made by the authors). It could be the case that attentional mechanisms (e.g., the monitoring of multiple players) is improved after Neurotracker training. Such a monitoring, however, is not only required for passing but also for dribbling (to avoid another player from taking the ball). Recommending this “training effect” to soccer coaches seems inadequate with the explained limitations. A replication of these effects in a study with a larger sample size, more objective assessments, and a fair control task should be presented first – the choice to collapse the passive and active control groups was questionable. It was not reported whether the results would have been significant without this post-hoc decision.

### Intervention studies not meeting the minimum quality criteria

The following studies are meant to be intervention studies but either have no control group at all, a control group that also received Neurotracker training, and/or no transfer test (see Table 4). Thus, they cannot provide evidence for any transfer, but they will be summarized here to provide a complete overview of the literature.

#### Study 10

Ten sessions of Neurotracker training might improve attention, visual information processing speed, and working memory and seems to change resting-state neuroelectric activity (Parsons et al., 2014). In this study, ten university students underwent Neurotracker training over 5 weeks and another ten students acted as a passive control group. Before and after the training, the authors administered 13 cognitive tests thought to be related to selective attention, divided attention, inhibition, short-term memory, working memory, and/or information processing speed. This is the only intervention study included in this review for which the significance level was set to .01. This decision was not preregistered and multiple improvements in the control group yielded *p* values just above .01. Therefore, to provide information more comparable to the other studies reviewed, we provide an overview of effects statistically significant in the intervention group but not in the control group if the significance level were set to .05 (see Table 6). The two groups were not well balanced for the cognitive variables and even in cases of statistically significant differences, they sometimes show similar scores in the post-test. For example, in the d2 test of attention (working memory), the training group had much lower pre-test scores than the control group (438 vs. 465) and both groups have similar values in the post-test (498 vs. 509). Due to these pre-test differences, only the improvements in the intervention group become significant. A similar concern is evident for the D-KEFS inhibition scores, where both groups have similar values in pre- (43.8 vs. 44.10) and post-test (38.4 vs. 40.2) and for D-KEFS Color Naming, where the control group began with lower values in pre-test (27.3 vs. 24.9) and the two groups have similar values after post-test (23.6 vs. 24.4). Instead of t-tests, a better approach might be an ANCOVA, treating pre-test as a covariate and post-test as the DV, as it would take into account the between-group pre-test differences. It should also be noted that, again, there was no correction for multiple comparisons for the 13 paired t-tests for the pre- and post-test. Another methodological concern is the possibility of ceiling effects in the “IVA+Plus” scores. When considering tasks without these methodological concerns, only an improvement in working memory remains.

The authors also present some EEG results indicating a decrease in 2–11 Hz slow-wave activity and a relative increase in beta waves which is interpreted as “attention benefits.” This interpretation is based on EEG studies with clinical trials and patient populations. Whether these findings for patients are transferrable to healthy students could be questioned. This study unfortunately uses unwarranted terms like “cognitive enhancer” to describe Neurotracker, which is not really appropriate given the limitations of the study.

#### Study 11

Legault et al. (2013) found that Neurotracker performance in young and old adults increases with practice. In this study, 20 younger adults (18–35 years old) and 20 older adults (64–73 years old) received five Neurotracker sessions (one every week). Both groups improved, but transfer was not assessed. Younger adults had higher speed thresholds (when tracking three and four targets) than older adults. Unfortunately, the paper does not mention whether any participants in the older adults group participated also in the study by Legault and Faubert (2012). The age range was reported to be between 64 and 73 years for both studies, and no note about participants’ Neurotracker experience is in either paper, so it is unclear whether the same participants took part in the study or that the same data was used in both studies (compare characteristics of older adults in study 6 – Table 5 with the group of older adults in study 11 – Table 6).

**Table 5:** Neurotracker intervention studies that include transfer tasks and at least one control group (i.e., a group that did not train with Neurotracker)

Study ID	Intervention group						Control group								Transfer
	N	Participant type	Age *M* or range	Training task	Training time (min)	Intervention interval	Type of control	Matched control	N	Participant type	Age *M* or range	Training task	Training time (min)	Training interval	Test
1	43	Students with ASD, ADHD, Intellectual disability	13	NT	105	15 sessions over 5 weeks	Active, passive	Age, FSIQ, PRI, WASI-II scores	86	students with ASD, ADHD, Intellectual disability	13	Computer game (2048)	105	15 sessions over 5 weeks	
2	63	Students	23	NT	90 or 150	3 or 5 sessions	Passive	-	21	students	23	-	0	-	EF
3	31	Wrestling, handball, biathlon, and alpine skiing	17-35	NT	186	9 - 76 sessions (M=26.5)	Passive	Age	29	soccer, paralympic sports, boxing orienteering	17-35	-	193	14 - 61 sessions (M=27.5)	WM, DM
4	25	Older adults	61-89	NT	420	14 sessions over 7 weeks	Passive	Education, dementia, memory, age	22	older adults	60-90	-	0	-	EF, DM
5	22	Volleyball experts	17	volleyball training + NT	384	8 weeks, 2 x 3 sessions each week	Active	Expertise and age	21	volleyball experts	21	volleyball training	384	8 weeks, 2 x 3 sessions each week	EF, WM
6	14	Older adults	64-73	NT	150	5 sessions, one each week	Active passive	age	27	older adults	64-73	Contrast task	n. d.	5 sessions	A, WM, PS,DM
7	13	Canadian Armed Forces	26-50	NT	100	10 sessions in 2 weeks	Active, passive	Demographic and cognitive variables	28	Canadian Armed Forces	21-50	dual n-back task (auditory + visual)	100	10 sessions in 2 weeks	A
8	18	Students	23	NT	100	5 sessions	Passive	-	18	students	23	-	0	-	WM
9	7	University-level soccer players	22	NT	210	5 weeks, 2 sessions each week	Active, passive	-	12	university-level soccer players	22	watch 3D soccer videos	150	5 weeks, 2 sessions each week	DM
10	10	Students	24	NT	600	10 sessions; 2 per week, over 5 weeks	Passive	Age, education	10	students	23	-	0	-	A, WM, PS

*Note.* Studies are sorted by the number of participants in descending order. Abbreviations: f = female; FSIQ = Full Scale Intelligence Quotient; mTBI = mild traumatic brain injury; NT = Neurotracker; PCS = post-concussion syndrome; PRI = Perceptual Reasoning Index; WASI-II = Wechsler Abbreviated Scale of Intelligence – Second Edition), EF = Executive functions, WM = Working memory, DM = Decision Making, A = Attention, PS = Processing speed

**Table 6:** Neurotracker intervention studies without control groups or with control groups also receiving Neurotracker training and/or studies with no transfer task

Study ID	Intervention group						Control group								Transfer
	N	Participant type	Age M or range	Training task	Training time (min)	Intervention interval	Type of control	Matched control	N	Participant type	Age M or range	Training task	Training time (min)	Control interval	Test
11	20	Younger observers	18-35	NT	150	5 sessions (one each week)	Active	-	20	Older observers	64-73	NT	150	5 sessions	-
12 (Exp.1)	16	badminton athletes;	23	NT + motor task	270	9 sessions	Active	-	13	Badminton athletes	23	Isolated MOT or motor task	270	9 sessions	-
12 (Exp.2)	13	Non-athletes	23	NT + perceptual task	270	9 sessions	Active	-	13	University-level athletes;	21	Isolated MOT or perceptual task	270	9 sessions	-
13	13	mTBI patients	14	NT	144	6 sessions	Active	-	13	No mTBI symptoms	13	NT	144	6 sessions	-
14	10	Clinically recovered mTBI patients	15	NT	90	6 visits (3 sessions each), one every 3-7 days	Active	age	10	Healthy control group of children	13	NT	90	6 visits (3 sessions each), one every 3-7 days	WM, PS
15	9	Children with PCS	9	NT	126	6 sessions; one every 2-7 days	-	-	-	-	-	-	-	-	WM, PS
16	1	Old man	80	NT + WM task	672	32 sessions over 16 weeks		-	-	-	-	-	-	-	WM, PS

*Note.* Studies are sorted by the number of participants in descending order. Abbreviations: f = female; FSIQ = Full Scale Intelligence Quotient; mTBI = mild traumatic brain injury; NT = Neurotracker; PCS = post-concussion syndrome; PRI = Perceptual Reasoning Index; WASI-II = Wechsler Abbreviated Scale of Intelligence – Second Edition), WM = Working memory, PS = Processing speed

#### Study 12

Consolidated Neurotracker training (i.e., Neurotracker training that is finished before another training task begins) seems to affect decision-making in a motor but not in a perceptual dual-task (Romeas et al., 2019). In two experiments, the costs of performing two tasks simultaneously were assessed when Neurotracker was combined with a motor task (Experiment 1) or a perceptual task (Experiment 2). In Experiment 1, 29 university badminton athletes were randomly assigned to four groups: group 1: simultaneous Neurotracker + motor task; group 2: consolidated Neurotracker + delayed motor task; group 3: isolated Neurotracker; group 4: isolated motor task. The motor task was a “birdie interception task” which required participants to intercept a virtual birdie with a badminton racket. The results indicated that Neurotracker performance (speed-thresholds) is impaired in dual-task situations in all groups but that all groups are able to improve single and dual-task performance with training. After the last training session, the highest scores were observed for group 2 (consolidated Neurotracker). In Experiment 2, 26 young adults (non-athletes) were randomly allocated to the same four groups as in Experiment 1. This time the secondary task was a biological-motion perception task, as also used by Legault et al. (2013). This task requires judgement of the walking direction (right or left) of a point-light walker positioned at a (virtual) distance of 4 m from the observer. Again, single- and dual-task performance improved with training. This time, there was no advantage of the consolidation group over the other groups. To control for potential sports expertise effects between the two experiments, 16 university level athletes received dual-task training with Neurotracker and the perceptual task. This athlete group and the non-athlete group from Experiment 2 showed no differences in performance. This study suggests that the addition of a motor task leads to greater interference with the Neurotracker as compared to a secondary perceptual task. Nevertheless, the results should be interpreted with caution as (a) the groups in Experiment 1 and 2 included only between five and eight participants, (b) there were speed-accuracy tradeoffs in the perceptual task in Experiment 2, and (c) there were ceiling effects in response accuracy for the perceptual task. Also unfortunate is that although the study did not investigate any transfer effects, specific implementations for training (to combine sport-specific tasks with Neurotracker) are suggested. The authors conclude that “[…] this study provides important insights into optimal training regimens” (Romeas et al., 2019, p. 944). This recommendation seems inappropriate as studies investigating potential transfer effects from dual-task training should be conducted first.

#### Study 13

Patients with a concussion history (mTBI) had similar gains after Neurotracker practice as healthy controls (Corbin-Berrigan et al., 2018). Two groups, an mTBI group (mean age: 16 years) and a healthy control group (mean age: 13 years), practiced Neurotracker for six sessions. Both groups improved but the control group showed higher training gains at session 2. The authors mention study limitations like sample size, age differences and a variable time since the occurrence of the injury. What is also noteworthy is that there were no between-group differences for absolute speed thresholds but only for normalized speed thresholds. Since normalized speed-thresholds have not always been reported in Neurotracker studies, a comparison between studies becomes very difficult; a point that we will discuss later.

#### Study 14

Like study 13, this study found that Neurotracker performance improved with practice in children with mTBI history as well as for a control population, with no significant changes in clinical measures (Corbin-Berrigan, Kowalski, Faubert, Christie, & Gagnon, 2020b). Ten participants in each, the intervention and control group, practiced Neurotracker in six sessions (visits every 3–7 days). A clinical test battery called ImPACT to assess balance, coordination, self-efficacy, fatigue, and memory was used. The ImPACT consists of six subscales: symptom checklist, verbal memory, visual memory, reaction time, processing speed, and impulse control (Corbin-Berrigan, Faubert, & Gagnon, 2020a). While both groups improved their Neurotracker performance over time by a similar amount, the clinical measures did not improve significantly for either group.

#### Study 15

Corbin-Berrigan, Faubert, and Gagnon (2020b) found no evidence for near transfer to cognitive and motor tasks for mTBI patients performing Neurotracker training. In their study, children with PCS had six sessions of Neurotracker practice. In every session, participants’ PCS symptoms were checked and cognitive efficiency (ImPACT; includes tests for verbal memory, visual memory, reaction time, processing speed, and impulse control) as well as balance control was tested, and quality of life was assessed with a questionnaire (PedsQL). Participants improved their Neurotracker performance, although looking at improvements between single sessions, participants only improved their performance from visit 3 to visit 4, which is also the time when symptom reductions were observed. In terms of transfer effects, there was no significant improvement in cognitive efficiency (*p* = .08, *d* = 1.37) or balance control (*p* = .11, *d* =.5). The only significant improvements were observed in “self-reported multidimensional fatigue” from the quality-of-life questionnaire. Methodological concerns include: absence of a control group and no correction for multiple comparisons for the 13 paired t-tests for the first and last visit. Moreover, participants underwent active rehabilitation in parallel which might have contributed to the results.

#### Study 16

Assed et al. (2016) conducted a single-case study with an 80-year-old man with dementia. He received Neurotracker and memory training in 32 sessions (two weekly sessions of 90 min each). Working memory training consisted of “verbal and visual mnemonic methods,” requiring to remember specific contents. Improvements that occurred for tracking one, two and three targets were claimed, although no statistical tests (or even error bars) were reported. Results indicate that training resulted in a near transfer, i.e., improved working memory performance (accuracy and speed), which was tested with a “computerized memory test” not used in training. Such improvements should, however, be interpreted cautiously, because it was a combination of memory and Neurotracker training and there was only one participant and no control group. Therefore, the results could be due to a placebo effect (Simons et al., 2016).

#### Summary of intervention study results

For the 16 published intervention studies, it was consistently found that people (athletes, students, healthy young and old adults, military, children with mTBI, children with neurodevelopmental disorders) improve their Neurotracker performance with practice. When it comes to transfer effects, however, any benefits of Neurotracker training are not yet clear (see Table 4).

Only three intervention studies investigated tasks designed to assess far transfer to real-world demands (see Table 4, “Far transfer effects”). Two of them found that Neurotracker training did *not* improve performance, one in a volleyball-specific task (Fleddermann et al., 2019) and one in a driving task (Harris et al., 2020b). Neither of these studies are included in the Neurotracker homepage. The only study cited there found that Neurotracker training improves passing decision making in soccer (Romeas et al., 2016). As explained earlier, however, this finding needs to be replicated with a larger sample, more objective assessments, and a theoretical account of why Neurotracker practice is expected to improve passing accuracy specifically (rather than other football-specific skills).

When looking at studies aiming at near transfer effects, six studies are not relevant because they had no control group or no transfer test (Table 4, grey font). From the ten studies that do provide evidence, nine assessed near-transfer effects, and eight found at least one positive near-transfer effect, but four of those had serious methodological limitations.

The only task type for which all near-transfer assessments found a benefit was attention. More specifically, Legault and Faubert (2012) found that the capacity to process biological motion improved, Parsons et al. (2014) and Fledderman et al. (2019) found an improvement in responding selectively to particular letters or digits. None of the studies were preregistered, however, and the first two had some additional methodological concerns (see evaluation of single studies above).

The most commonly assessed transfer effect was working memory. From the six studies on this attentional skill, five found a positive effect of Neurotracker practice, but none were preregistered, three had additional methodological issues, and one study showed no effect.

A positive transfer effect was reported for connecting numbers on paper in order with a pencil in athletes (Fleddermann et al., 2019). Clear beneficial Neurotracker transfer effects for executive functions (here: inhibition) have only been found in one study (Tullo et al., 2018b). There was no transfer effect of Neurotracker training on any of the executive-function tests of the Delis–Kaplan Executive Function System (D-KEFS) in older adults (Musteata et al., 2019). Overall, whether Neurotracker training improves attentional skills is unclear because there are a number of methodological concerns in intervention studies investigating near-transfer effects as well as null results and possible publication bias.

According to the Neurotracker website, Neurotracker training improves awareness, but no published study appears to have investigated whether Neurotracker training might improve awareness or perception.

It should be noted that even studies examining similar populations with the same methods could either not replicate their working memory transfer effects in a second study in student populations (Harris et al., 2020a, b) or find any effect in in patients with a concussion history (Corbin-Berrigan, Kowalski, et al., 2020b; Corbin-Berrigan, Faubert, & Gagnon, 2020a). This adds to the concern that the best evidence (best because the study included an active and passive control group) for a working-memory transfer effect, found in military populations with 10 sessions of Neurotracker training (Vartanian et al., 2016), may not replicate.

### Overall summary I: Critical remarks on the methods of Neurotracker studies

In this section, we summarize some methodological concerns in Neurotracker papers and relate them, if possible, to existing MOT research, to explain the impact of these issues on results. We hope that this list will lead to improved methodology in future Neurotracker research.

1. For the statistical analyses, p-values should use the standard .05 threshold (or fully explain and preregister why they use a different standard) and be corrected in case of multiple post-hoc pairwise comparisons. The standardization issue was a problem in the study by Parsons et al. (2014) where p-values were only reported as being significant with *p* < .01. This can evoke suspicion that this threshold was chosen to conceal the many effects found for the control group with *p* < .05 but larger than *p* < .01. The issue of the failure to address multiple comparisons was observed in a number of studies (Corbin-Berrigan, Faubert, & Gagnon, 2020a; Musteata et al., 2019; Parsons et al., 2014). These studies had a large number of post-hoc comparisons with *p*-values being close to .05. These “effects” would have probably not been statistically significant, if the *p*-values had been corrected for multiple comparisons.
2. Studies must be preregistered for readers to have confidence in the p-values associated with the statistical tests, as they may otherwise be diluted by practices including p-hacking, change of analysis strategies (e.g., Romeas et al., 2016), and optional stopping rather than a priori planned sample sizes (Nosek, Ebersole, DeHaven, & Mellor, 2018). For example, not committing to a specific sample size or stopping rule when beginning a study can inflate effect sizes or create significant effects that are spurious (Simmons et al., 2011). Unfortunately, none of the Neurotracker studies appear to have been preregistered.
3. Many studies describe significant improvements within the training group, but neglect reporting differential improvements (Legault & Faubert, 2012) or rely on an omnibus interaction test without contrasting an individual group with the control group (Harris et al., 2020b). A difference in significance between the training and control group (i.e., one is <.05 and the other is not) is not the same as a significant difference in the *extent* of improvement. Unless the study showed that the improvement on the transfer task in the Neurotracker group was significantly greater than the improvement in the control group, it did not provide evidence for transfer of training. Besides interaction tests, another approach is that of Tullo et al. (2018b), who used pre-post differences in inhibition skills as their dependent variable.
4. It is not clear whether participants from one study also took part in another study. As an example, the studies by Legault and Faubert (2012) and Legault et al. (2013) both used older adults and reported the same age range (64–73 years old). It was not mentioned whether any of the participants had experience with Neurotracker. Similarly, Faubert and Sidebottom (2012) and Faubert (2013) both report data from an English Premier Team club, a hockey team from the National Hockey League, and a rugby team from the European Rugby. Again, there is no indication whether the same athletes were in both data sets.
5. A variety of stimulus characteristics have been used in Neurotracker studies. In some studies the objects bounce off the walls and also change direction when they are close to other objects (Legault et al., 2013; Legault & Faubert, 2012; Romeas et al., 2016). In another study, objects only bounce off the walls but not off other objects (Harris et al., 2020a) and in other studies, it is not mentioned how objects interact (Assed et al., 2016; Moen et al., 2018; Vartanian et al., 2016). This is an important issue as previous MOT research has shown that object distances and interactions affect attentional (Iordanescu et al., 2009; Shim et al., 2008) and perceptual strategies (Vater et al., 2017b; Zelinsky & Todor, 2010) as well as tracking performance (Holcombe et al., 2014; Vater et al., 2017b). Related to this issue, objects randomly changed direction at times in some Neurotracker studies (Moen et al., 2018; Tullo, Faubert, & Bertone, 2018a, b) but remained on straight paths in others (Musteata et al., 2019; Romeas et al., 2016). Objects with random motion trajectories can be more difficult to track and require a greater amount of sustained attention, although this may be less true when there are more objects to track, because participants then seem to have less knowledge of object velocity (Horowitz & Cohen, 2010; Howe & Holcombe, 2012; Luu & Howe, 2015).
6. The number of targets to track varied between studies. While most studies used four targets and four distractors, in others only three out of eight objects needed to be tracked (Fabri et al., 2017; Mejane et al., 2019; Plourde et al., 2017; Tullo et al., 2018b) and others used multiple conditions (e.g., Assed et al., 2016; Moen et al., 2018; Tullo, Faubert, & Bertone, 2018a; Tullo et al., 2018b). The more targets have to be tracked, the more difficult the task (Meyerhoff et al., 2017; Pylyshyn & Storm, 1988) and more distractors may mean tracking performance is more affected by suppression ability or by skills specific to target-distractor interactions. These differences also presumably impact on the absolute and normalized speed thresholds and complicate a comparison between studies. One possibility, for example, is that with more targets, visual short-term memory capacity and skill at switching attention among targets are both more critical to performance (Lovett et al., 2019).
7. Different dependent variables were used. Some studies used absolute speed thresholds (Corbin-Berrigan, Faubert, & Gagnon, 2020a; Fleddermann et al., 2019) while others used normalized speed thresholds (Corbin-Berrigan et al., 2018), and others reported the average speed at which participants successfully tracked three target spheres (Tullo, Faubert, & Bertone, 2018a; Tullo et al., 2018b), the absolute speed gain (Corbin-Berrigan et al., 2020b), or the number of correct responses (Fabri et al., 2017). To better compare results across studies, normalized speed thresholds may best capture improvement differences between groups, because pre-test values are taken as the baseline.
8. Standardization of, or at least detailed reporting of, display sizes and object spacing. Different Neurotracker studies used images projected on multiple walls of a room with shutter glasses to create 3D spheres (Legault et al., 2013; Legault & Faubert, 2012), a single screen with 3D glasses (e.g., Lysenko-Martin et al., 2020; Vartanian et al., 2016) head-mounted displays (Romeas et al., 2016; Tullo, Faubert, & Bertone, 2018a), and tablet devices (Chermann et al., 2018; Harris et al., 2020b). Not all of the studies mention the size of the visual field, which is unfortunate because the distance into the periphery of the objects has a very large effect on acuity (Strasburger et al., 2011), causing perceptual demands to vary, potentially dramatically. Most studies that do mention the size indicate it to be between 42° and 48° visual angle (e.g., Chermann et al., 2018; Faubert, 2013; Fleddermann et al., 2019; Harris et al., 2020a; Legault & Faubert, 2012; Mangine et al., 2014; Romeas et al., 2019).
  Even when the maximum distance into the periphery of the objects is equated, the spacing of the objects has a substantial effect on tracking performance, and on which processes limit performance (Intriligator & Cavanagh, 2001). Denser spacing results in both greater attentional demands and a greater effect of perceptual constraints (Holcombe et al., 2014; Tombu & Seiffert, 2011), and the “crowding” perceptual constraint is known to differ substantially across participants (Greenwood et al., 2017) and training may change it significantly (Bertoni et al., 2019). The Neurotracker training studies with different object spacings may, then, be studying the training of different skills.
9. Studies used different types of responses to analyze tracking accuracy. Studies varied in whether they required a verbal response (e.g., Musteata et al., 2019; Parsons et al., 2014) or a key or button response (e.g., Moen et al., 2018). It cannot be ruled out that these two response modalities place different demands on working memory, in part because they result in different amounts of total time to indicate all the objects.

### Overall summary II: Factors that complicate the comparison of intervention studies

Many Neurotracker studies had inadequate control groups and control group tasks. From the 16 interventions, only nine included an active control group (three of these included both an active and a passive control group, (Romeas et al., 2016; Tullo et al., 2018b; Vartanian et al., 2016), five only had a passive control group (Harris et al., 2020a, b; Moen et al., 2018; Musteata et al., 2019; Parsons et al., 2014), and two had no control group (Assed et al., 2016; Corbin-Berrigan et al., 2020b). Unfortunately, some of the control groups had substantially different scores on primary outcomes even prior to the intervention (e.g., Harris et al., 2020a, b) complicating interpretation.

Many intervention studies had small sample sizes, making the results unreliable. Some of the intervention groups consisted of ten or fewer participants (Assed et al., 2016; Corbin-Berrigan et al., 2020b; 2020; Parsons et al., 2014; Romeas et al., 2016, 2019). The resulting low statistical power means that we cannot know whether contradictory results are due to statistical fluctuations or sometimes-small discrepancies in methods (see Corbin-Berrigan et al., 2020b, and Corbin-Berrigan, Faubert, & Gagnon, 2020a).

Published studies had a variable amount of training sessions and times between training sessions *within* studies. In some studies, the amount of training sessions is not constant for all participants. In the study by Faubert (2013, p. 3) the observers trained “[…] up to 15 sessions separated over a minimum of five different days [….].” In a study by Moen et al. (2018), participants had between nine and 76 training times. The dose-response relation between training and effect is very difficult to discern with such a high variability. Moreover, if one group of participants receives more training than the other, comparing group performance becomes impossible. Another issue is the variable time interval between training sessions. In one study the authors mentioned that there were 3–7 days between sessions (Corbin-Berrigan et al., 2020b). While it may not always be possible to guarantee constant intervals between sessions, especially not in clinical populations receiving a treatment or in sports where athletes have a dense training schedule, inter-training intervals should be equated between groups. Providing an explanation for the reason for a training protocol, which typically was not done, is important to make progress on refining or standardizing protocols.

Different studies used highly disparate amounts of training, varying from 6 ×3 sessions or approximately 90 min of training; (Corbin-Berrigan et al., 2020b; Harris et al., 2020b; Parsons et al., 2014) to 32 × 3 sessions or 672 min of training (Assed et al., 2016). Since some studies showed a trend for Neurotracker performance improvement of control groups that received only Neurotracker experience only in test sessions (Harris et al., 2020a, b), suggesting that improving Neurotracker performance requires little training. The same is true for healthy children and clinically recovered mTBI patients, who both show highest training gains from the first to the second training session (Corbin-Berrigan et al., 2020b). More important is for future studies to focus on what is required to find transfer effects in other tasks.

## Overall discussion and suggestions for future Neurotracker research

The reviewed studies indicate that some attentional skills like working memory, sustained attention, processing speed or inhibition might improve with Neurotracker training, but the evidence to date is weak. The findings are mixed and could be statistical false positives or the result of greater expectations (placebo effect) in the Neurotracker groups, even relative to some of the active control groups, for which expectations were never measured. There is a large variability not only in study designs but also in Neurotracker stimulus characteristics, experimental setups and statistical analyses.

What can sports coaches and players learn from our review? No clear improvements have been found for the all-important transfer of Neurotracker training to actual sports skills. The only positive far-transfer effects found were in a single study, for soccer pass decision-making but not other soccer decisions (Romeas et al., 2016). A preregistered replication with improved methods is needed before one can be confident in the result. In another study, Neurotracker training, in addition to regular training, did not lead to higher training gains in on-field performance (Fleddermann et al., 2019). Since practice time is typically valuable, coaches should carefully weigh the pros and cons of using any training tool. Presently, the evidence for far-transfer is too weak to justify replacing time available for sport-specific training with Neurotracker training.

What should researchers take into account in future studies? As discussed above, Neurotracker studies would benefit from being more theoretically driven and precisely targeted, using MOT research to predict how Neurotracker training might improve attentional skills and guide the choice of task parameters. Basic studies can assess whether the supposedly trained cognitive skills are in fact taxed or “overloaded.” To better combine the results across multiple Neurotracker studies, the methods (i.e., experimental setup, instructions, responses, analysis) need to be described in full detail to allow other researchers to replicate findings. Preregistration of study designs and the use of more appropriate statistics (e.g., corrected post hoc comparisons) would reduce the concern that the transfer effects found may be statistical false positives. More confidence in the findings would also result if researchers shared the (anonymized) data of the studies, as that allows confirmation that there were no statistical errors (one study of psychology articles found that nearly half made a particular kind of error Green et al. (2018), and better enables meta-analysis to compare studies using common statistical methods.

A particular focus of future research should be the attentional skill sometimes known as “awareness.” No intervention study has yet investigated if awareness can be trained with Neurotracker. To investigate this in future studies, eye-tracking methods could reveal, whether strategies such as for the use of peripheral vision develop with Neurotracker training and if these transfer to real-life tasks, for example, to soccer situations. It comes as a surprise that no published Neurotracker study has yet used eye-tracking to determine how participants are using peripheral vision. Participants often are instructed to use peripheral vision to monitor target movements, but it remains unclear whether they follow these instructions. Since MOT research has found that gaze strategies can explain performance differences and that stimulus characteristics affect perceptual strategies (e.g., Fehd & Seiffert, 2010; Vater et al., 2017b; Zelinsky & Neider, 2008; Zelinsky & Todor, 2010), it seems mandatory to control for (a) eye movements and (b) stimulus characteristics. If peripheral vision usage is indeed linked to better Neurotracker performance, this would be a strong argument for a prediction that especially game sports could benefit from Neurotracker training, because peripheral vision is known to be important here (c.f., Vater et al., 2020).

What can the manufacturer take from our review? The main aims of a company typically are to sell products and potentially access the training data of customers to improve their devices. In contrast, the customers, for example professional sports clubs, are primarily interested in improving the performance of their athletes. If the manufacturer shows positive training effects in their research, sports clubs will likely invest in such training devices, which sounds like a perfect fit between the two parties. From an objective research perspective, however, promising positive skill transfer with little or mixed research evidence is not appropriate. Therefore, Neurotracker research results should be communicated more transparently, with fewer claims that appear to exaggerate or go beyond the evidence. For example, the Neurotracker’s webpage claims that Neurotracker training helps to “stay sharp under time pressure,” “perceptually slow down the environment,” or “avoid overly impulsive action” have not been specifically researched. The Neurotracker webpage reference list provides a biased picture of the evidence by omitting certain studies with null results.

We agree with the statement of Simons et al. (2016, p. 173) that “If a company claims scientific proof for the benefits of its products, it must adhere to best scientific practices.” Based on the current status of Neurotracker research, promoting it as a training tool for professional sports or other domains to improve real-world perceptual-cognitive skills is premature because the far-transfer effects are up to now either not there or not very solid. Even the claims of near transfer of Neurotracker training to attentional skills are questionable given the many methodological concerns in published studies. With our review, we hope to have shown how sport science and basic science can be better utilized to guide research to assess which skills can be improved with Neurotracker training.

## Supplementary Information

ESM 1 (DOCX 83 kb)

## Footnotes

1 https://docs.google.com/spreadsheets/d/1tdEChpYHH1nnTc2Chqow0FY7Rj70jHrbLl5OhOPmQbE/edit#gid=0
