# Supplementary material for: A critical systematic review of the Neurotracker perceptual-cognitive training tool
Source: Psychon Bull Rev. 2021 Apr 5;28(5):1458–83. doi: 10.3758/s13423-021-01892-2 (PMC8500884; doi:10.3758/s13423-021-01892-2)
Supplement: Supplementary file 1 — (DOCX 83 kb) [file 13423_2021_1892_MOESM1_ESM.docx]

# Supplement

## Summary of Non-intervention Studies

In an observational study, Harenberg et al. (2016, p. 387) expected that Neurotracker performance would correlate positively with simulated surgical performance because “it requires attention skills that are important when performing surgery”. In their regression models, Neurotracker scores were indeed related to surgery skills and explained 29% of the variance for time to task completion and 28% of the surgical arm movements. Mangine et al. (2014) related basketball-specific measures of performance to Neurotracker performance (tracking speed and reaction time) in twelve players. The authors found that tracking speed was strongly associated with statistics from game play including assists (*r* = 0.78; *p* = 0.003) and steals (*r* = 0.77; *p* = 0.003) but there was no significant relationship with game statistics for a second task of simple visuomotor response time. The authors conclude that the results reflect the “basketball player’s ability to see and respond to various stimuli on the basketball court” (Mangine et al., 2014, p. 2406). Note, however, that correlations based on only twelve participants are expected to be extremely unstable and have very low statistical power (Schönbrodt & Perugini, 2013). Michaels et al. (2017) compared older and younger drivers on driving measures (e.g., crash rate, mean speed) and Neurotracker performance. Lower Neurotracker performance was significantly associated with an elevated crash risk (*r*(113) = -.31, *p* < .001) and a lower average speed (*r*(113) = .47, *p* < .001). Because no other basic cognitive or attentional tasks were used (unless one counts the simple response time task of Mangine et al. (2014), it is unclear whether Neurotracker is more correlated with the skills of interest than any other standardized cognitive or attentional task.

Tullo, Faubert, and Bertone (2018), found that MOT performance positively correlated with fluid-reasoning intelligence (*r* = .41, *p* = .045) and that adults with a high fluid reasoning IQ had better Neurotracker performance. It should be noted that although like other studies it was not preregistered, it has a relatively high quality as the methods are clearly described, the findings are related to a number of MOT studies and there is a detailed critical evaluation of study limitations. Finally, Lysenko-Martin et al. (2020) compared different age groups with and without a history of concussions. The aim of the study was to test whether 3D-MOT is sensitive to cognition and balance measures used to diagnose the post-concussion syndrome (PCS). They found that Neurotracker performance of 104 under 13 year-olds with a history of concussions was not impaired but was very weakly associated with cognition (*r* = .12, *p* < .001) and balance (*r* = .04, *p* = .02). An important note for this study is that cognitive functioning was better for females than males but that Neurotracker performance was higher for males than females.

Using a within-between-group design, Chermann et al. (2018) tested healthy rugby players and rugby players with concussions. The results indicated better performance for healthy players, improved performance in both groups after training and significantly better Neurotracker performance in the concussed group at the time they returned to play compared to 48h post-concussion assessment. Plourde et al. (2017) compared Neurotracker performance with and without stereoscopic vision (3D glasses) for children, adults and older adults. Neurotracker performance was best for adults, followed by children and older adults. Performance was better with compared to without stereoscopic vision in adults and children but not in older adults. A correlation between scores in a standardized stereoacuity test and Neurotracker scores was not significant for older adults, suggesting that the two tasks do not measure the same stereoscopic processing (correlations for the other groups were not possible as they reached the maximum vision acuity scores). In the third study of this category, Fabri et al. (2017) compared two healthy age groups (5–11 years and 12-18 years) under single-task (Neurotracker), and dual-task (postural stability task and Neurotracker) conditions on either a firm or less stable foam surface. The older group showed better Neurotracker performance than younger participants on both surfaces. In both age groups, postural stability but not Neurotracker performance was impaired in dual-task situations. As a side note, there were no differences in Neurotracker performance between male and female participants, in contrast to the results reported by Lysenko-Martin et al., 2020). Legault et al. (2013) compared Neurotracker speed-thresholds of younger (22-34 years) and older (61-74 years) participants, and found better performance for the younger participants.

In a within-group design, Mejane et al. (2019) analyzed knee-joint kinematics in single task (jumping and landing trials) and dual-task (jumping and landing + Neurotracker) conditions. Between two sessions, both with single- and dual-task conditions, participants underwent a muscular fatigue protocol. It was found that the addition of the Neurotracker task had no significant effect on any knee rotations, either pre- or post-fatigue.

In a between-group design, Faubert (2013) compared Neurotracker learning rates for groups with different levels of sports expertise for a large sample (n = 308). The paper concluded that “professionals as a group dramatically differ from high-level amateur athletes, who dramatically differ from non-athlete university students”. (Faubert, 2013, p. 1153), but no statistical tests were done. Visual inspection of the learning rate plots suggested that professional athletes started with higher scores and learned fastest. It is notable that in classic MOT studies, an expert advantage could not always be shown (e.g., see Schwab & Memmert, 2012).

Chamoun et al. (2017) tested whether a pharmacological manipulation of cholinergic neurotransmission would enhance complex visual processing in healthy individuals. They compared a group receiving a medication against Alzheimer’s disease (donepezil) with a group receiving a placebo (lactose) over 5 weeks. The authors found similar Neurotracker learning effects in both groups in the final test. Fragala et al. (2014) investigated whether 6 weeks of resistance training in older adults improves Neurotracker performance. Indeed, the training group performed better than a passive control group.

Summing these results up, they indicate significant positive relationships between Neurotracker scores and task performance for a variety of skills, although none of these appear to have been pre-registered or replicated, so they should be treated with caution. The finding of older participants’ Neurotracker performance being associated with poorer driving performance seems discrepant with a finding by Bowers et al. (2013) of no relationship with a different MOT task). All of the relationships were simple correlations from observational studies, such that casual inferences are not warranted. For example, while the developers of Neurotracker claim that the reasons why expert athletes score better on Neurotracker than lesser skilled and non-athletes is because it taps into perceptual-cognitive skills used in competition, it is equally possible that this effect is due to selection (e.g., individuals with poorer vision choose not to pursue sports) and/or that higher scores on Neurotracker are due to general factors (e.g., athletes are more competitive and motivated).
